# Intermittency in phytoplankton bloom triggered by modulations in vertical stability

**DOI:** 10.1038/s41598-020-80331-z

**Published:** 2021-01-14

**Authors:** Madhavan Girijakumari Keerthi, Marina Lévy, Olivier Aumont

**Affiliations:** Sorbonne Université (CNRS/IRD/MNHN), LOCEAN-IPSL, Paris, France

**Keywords:** Biogeochemistry, Ocean sciences

## Abstract

Seasonal surface chlorophyll (SChl) blooms are very chaotic in nature, but traditional bloom paradigms have climbed out of these subseasonal variations. Here we highlight the leading order role of wind bursts, by conjoining two decades of satellite SChl with atmospheric reanalysis in the Northwestern Mediterranean Sea. We demonstrate that weekly SChl fluctuations are in phase with weekly changes in wind stress and net heat flux during the intial state of the bloom in winter and early spring, thus expanding the convection shutdown hypothesis of bloom onset to subseasonal timescales. We postulate that the mechanism reflected by this link is intermittency in vertical stability due to short-term episodes of calm weather in winter or to stormy conditions in early spring, leading to short-term variations in light exposure or to events of vertical dilution. This strong intermittency in phytoplankton bloom may probably have important consequences on carbon export and trophic web structure and should not be overlooked.

## Introduction

A striking characteristic of phytoplankton blooms are the fluctuating patterns in sea-surface Chlorophyll (SChl, a proxy for phytoplankton biomass) that punctuate the transition from low abundance in winter to biomass accumulation in spring^1–10^. The chaotic nature and varying intensity of these subseasonal events make each seasonal cycle unique. In some years, the temporal evolution of SChl deviates from the traditional pattern characterized by a single seasonal peak and exhibits a succession of several peaks^8^. These subseasonal events make an important contribution to bloom variability^8^ and are therefore an important factor for the development of the upper trophic levels of the food web^11^ as well as for the efficiency of the biological carbon pump^12^.

Traditional bloom onset paradigms have climbed out of these subseasonal variations by focusing on the strongest, or latest, peak. They relate the period of rapid growth of SChl between winter and spring to the change in vertical stability, and to the increased light exposure of the phytoplankton population associated with it. Under these models, a sustained bloom cannot start before a seasonal tipping point is met, such as a critical depth or critical mixing intensity^10,13–20^. This view is in conflict with the strong chaotic intermittency seen in SChl time series. Incidentally, evidences of intermittent near-surface phytoplankton accumulation during spells of calm weather in winter, with biomass being mixed down in the next storm, leading to a decrease in SChl until weather conditions improve again, were present in the data sets used to test spring bloom models^13,14^ but have been overlooked.

Here we explore the links between the surface bloom and vertical stability at intraseasonal timescales, using two decades of satellite SChl data and atmospheric reanalysis in the Northwestern Mediterranean Sea. Our focus on this region is motivated by a previous study that highlighted the strong intensity of subseasonal SChl fluctuations there^8^. At the seasonal time scale, Ferrari et al.^13^ showed that surface blooms in the North Atlantic were triggered by a change from cooling to heating in Net Heat Flux (NHF) at the end of winter, with a similar dataset. They argued that this change resulted in a rapid shutdown of vertical convection. The physical basis came from a preliminary numerical study which demonstrated that the reduction in air-sea fluxes at the end of winter could be used as an indicator of reduced turbulent mixing^10^. Our intention is to generalize this concept to all time scales from seasonal to subseasonal. Intermittency in turbulent mixing being primarily driven by intermittency in atmospheric forcing, we want to test whether subseasonal changes in SChl can be explained by subseasonal changes in atmospheric conditions.

The seasonal phenology of SChl in the Northwestern Mediterranean Sea is well documented^21^. As in the North Atlantic, it has been traditionally related to changes in the mixed-layer depth (MLD)^22,23^, with the SChl bloom starting as soon as the water column is more stable, and a time lag of about 1 month between the time of maximum MLD in winter and maximum SChl in spring. As a first step, we verified that the convection shutdown hypothesis of seasonal bloom initiation^13^ applied to this region. This hypothesis is easier to test and more precise than the critical depth hypothesis^14^ because the surface mixed-layer is not always associated with strong rates of turbulent mixing^1,24^. As a second step, we investigated the hypothesis that subseasonal modulations in vertical stability triggered by wind bursts (and reflected by temporal variations in NHF) explain subseasonal SChl variations during the initial states of the bloom in the Northwestern Mediterranean Sea. The paper ends with a discussion on uncertainties and wider implications of these results.

## Results

Our focus is on the winter (January–February) to spring (March–April) period, which covers the entire SChl bloom from its onset to its decay. This is also when storms are the most frequent and subseasonal variations in SChl are intense^8^. We first describe the main seasonal changes in SChl between winter and spring and relate them to changes in NHF. We then extend the analysis to subseasonal fluctuations.

**Figure 1:**
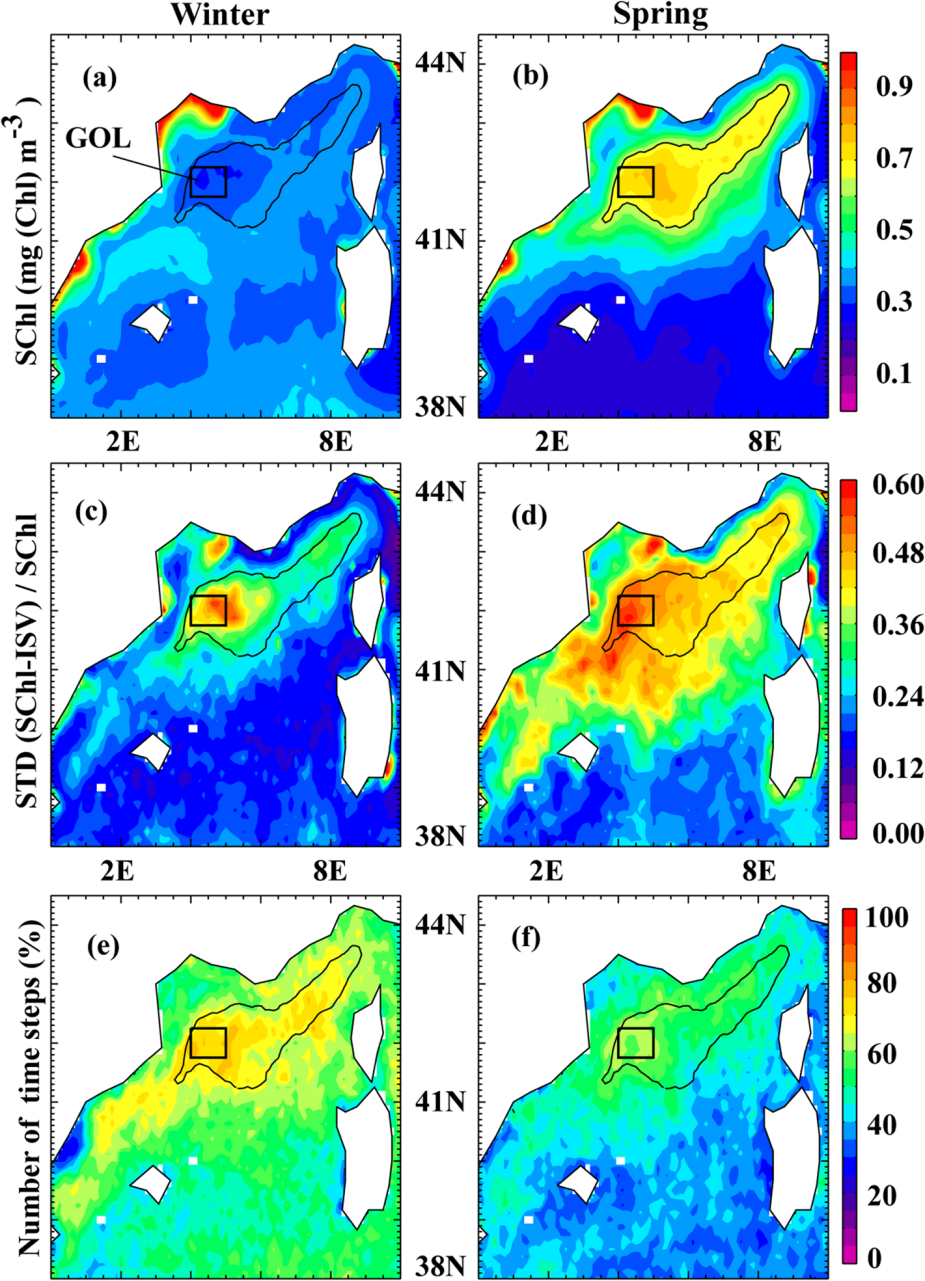
(**a**,**b**) Surface Chlorophyll climatology (SChl, in mg Chl m^−3^) in the Northwestern Mediterranean Sea in winter (January–February) and spring (March–April), over the period 1998–2017. (**c**,**d**) Standard deviation (STD) of intraseasonal SChl fluctuations (SChl-ISV) in winter (resp. spring), normalized by the mean SChl in winter (resp. spring). Intraseasonal SChl fluctuations were extracted from the total signal using the Census X-11 technique, following Keerthi et al.^8^. (**e**,**f**) Percentage of time steps for which the time derivative in Net Heat Flux and the SChl net growth rate have the same sign, during winter and during spring. In all panels, the black contour delimitates the bloom region, which we defined as the region where the climatological spring SChl is greater than 0.65 mg (Chl) m^−3^. The black square marks the Gulf of Lion (GOL) box.

The Northwestern Mediterranean Sea is one of the few regions in the world’s ocean where deep convection occurs^25^. During winter, a deep-mixed patch of dense, nutrient rich water is formed during intense mixing episodes and appears as a blue zone devoid of SChl in ocean color images^26^ (Fig. 1a). The air-sea heat budget shows a mean seasonal trend from strong buoyancy losses in winter to strong gains in spring, associated with warming. This trend drives seasonal stratification. Consequently, in spring, the pattern in SChl is the reverse figure of the winter pattern (Fig. 1b): the largest spring SChl values mirror the lowest SChl winter values, which delineate the convective area and the corresponding largest winter nutrient inputs^27^.

**Figure 2:**
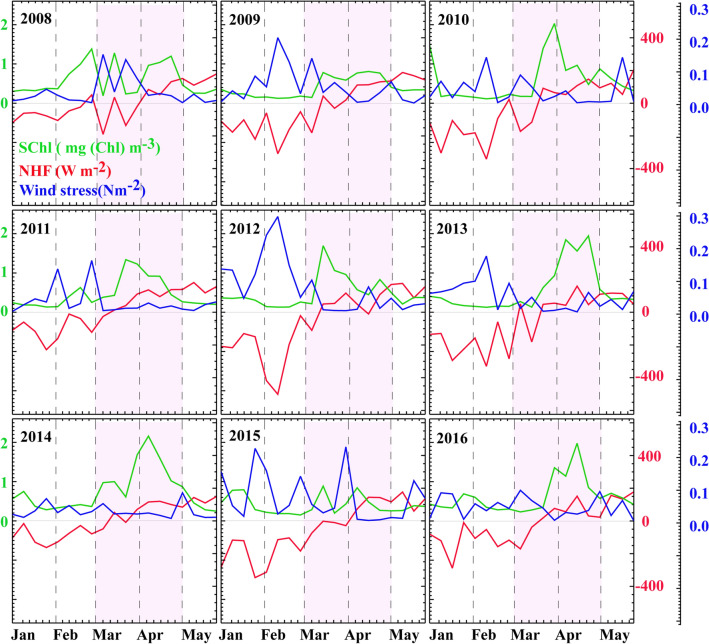
Timeseries of Surface Chlorophyll (SChl, green curves), Net Heat Flux (NHF, red curves) and wind stress (blue curves) averaged over the GOL box between January and May, from 2008 to 2016. The pink shading highlights the spring period (March–April).

SChl time series between winter and spring in the core of the bloom region between 2008 and 2016 provide a few examples of the annually repeating SChl spring bloom (GOL Box, Fig. 2). The complete time series from 1998 to 2017 is provided in Supplementary Fig. S1. SChl are generally lowest in February, increase sharply around mid-March and decrease in April. There are numerous exceptions to this general rule, and these will be discussed later. For now, we examine whether the main seasonal increase in SChl occurs concurrently with the change of sign in NHF, in support of the convection shut down hypothesis^13^.

**Figure 3:**
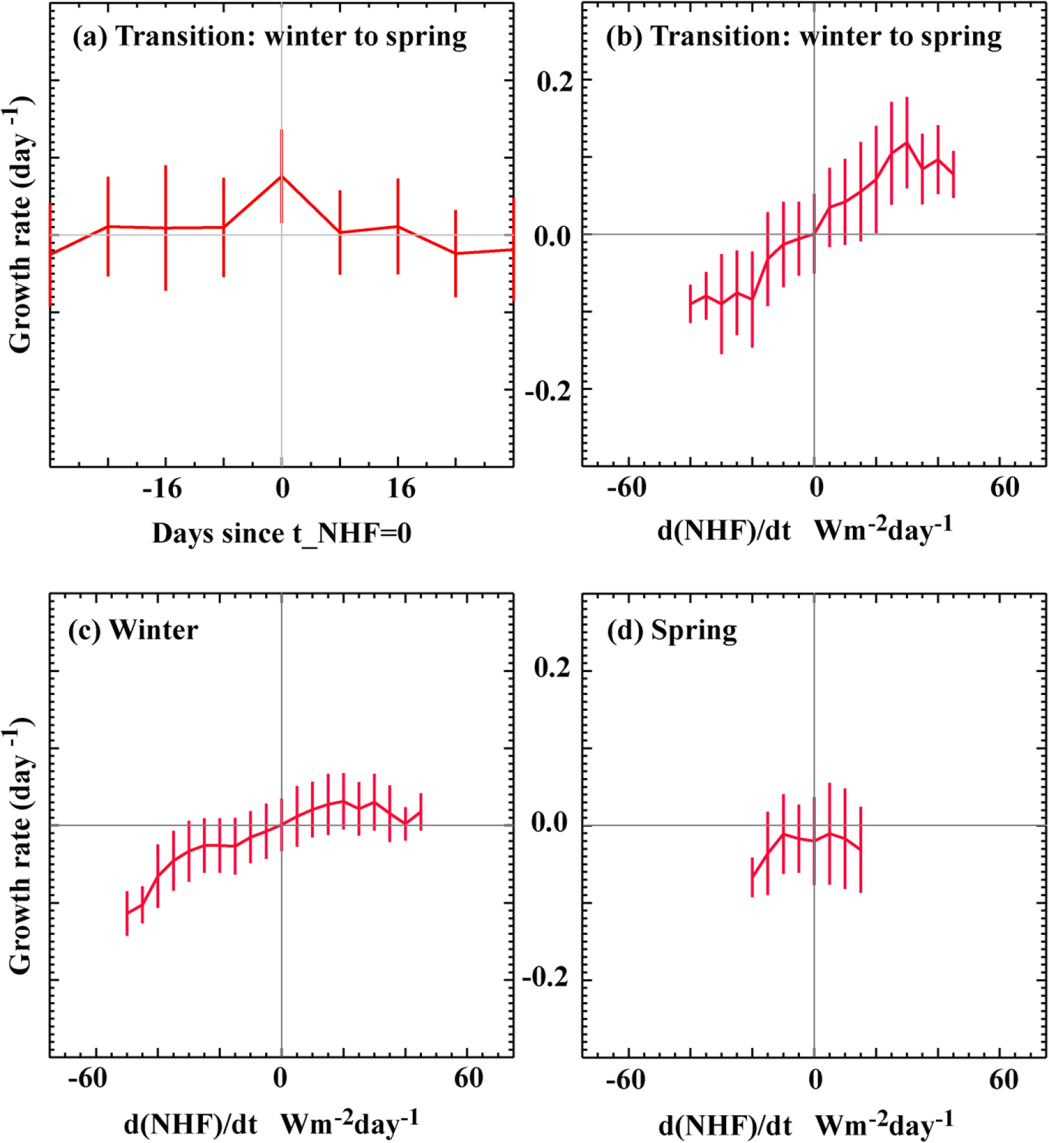
SChl net growth rate (in day^−1^) (**a**,**b**) during the winter to spring transition phase: when the NHF changes sign, (**c**) during the unstable winter phase: when NHF are initially negative and remain negative and (**d**) during the stable spring phase: when NHF is initially positive and remains positive. (**a**) SChl net growth rate is shown against time (in days) since the NHF has switched from negative to positive and has remained positive thereafter (t_NHF = 0). The mean net growth at t = 0 is larger than at any time step before (t = − 24d, − 16d, − 8d) or after (t = 8d, 16d, 24d). (**b**–**d**) SChl net growth rate against temporal changes in NHF. Positive (resp. negative) changes in NHF are used as a proxy for increased (resp. decreased) vertical stability. In winter and early spring, increased (resp. decreased) vertical stability at weekly time scale are associated with enhanced (resp. decreased) net growth rate. SChl Growth rates are computed for individual events, i.e. at each 8-day time step and at each 0.125° × 0.125° pixel in the bloom region over 1998–2017 within the time period January–April, and are then bin-averaged, with the vertical bars representing one standard deviation. Data of each single event before bin-averaging are shown in Supplementary Figure S2.

A general feature is that the NHF turns positive in mid-March and remains positive thereafter (Fig. 2 and Supplementary Fig. S1). This change of sign consistently coincides with a sharp increase in SChl. This is more quantitatively seen in Fig. 3a which shows the value of the SChl net growth rate at the time at which NHF turns and remains positive (t = 0) and at three consecutives 8-day periods before and after t = 0 (t = − 24d, − 16d, − 8d, 8d, 16d, 24d), over the entire time series (1998–2017) and for the entire bloom region. Averaged over all events, the net growth rate is ~ 0.1 day^−1^ at t = 0 but it is close to zero otherwise. Indeed, the net growth rate is always strictly positive (between 0 and 0.2 day^−1^) for each individual event at t = 0, whereas before and after t = 0, net growth rates are more equally distributed between positive and negative values. This result expands the results of Ferrari et al.^13^ to the Northwestern Mediterranean Sea. Incidentally, Fig. 3a also reveals large values of the standard deviation of the net growth rates, indicating large values of the net growth rate at times before and after t = 0, which we will examine thereafter.

Now we examine the subseasonal events that punctuate the mean seasonal evolution. These subseasonal variations have large intensity both during winter and during spring, although the spatial extent of the region where their intensity is large is more limited in winter than in spring (Fig. 1c,d). Subseasonal variations induce a large variety of typologies of the SChl evolution (Fig. 2 and Supplementary Fig. S1). The seasonal evolution of SChl seldom shows as a single period of rapid SChl accumulation in spring. There can be early periods of accumulation in February (2008, 2011), a series of two to three well separated periods of accumulation of similar magnitude during spring (2008, 2015), a main period of accumulation followed by (2010, 2012) or preceded by (2011, 2014) smaller peaks, or double-headed peaks (2009, 2013, 2016).

In order to explore the link between short term episodes of calm weather in winter or intensified mixing in spring with intraseasonal SChl fluctuations, we examined the relationship between the time derivative of the NHF and the net SChl growth rate, for each 8-day time step in winter and spring. The underlying assumption is that periods of reduced mixing during winter should correspond to less negative NHF (and lower wind stress), while periods of intensified mixing in spring correspond to lower NHF (and stronger wind stress). The comparison of NHF and wind stress (WS) time series (Fig. 2 and Supplementary Fig. S1) clearly reveals that subseasonal variations of NHF mirror those of the WS. One recurrent feature is that the seasonal increase in NHF is interrupted by storms that last for about one time step (i.e. less than a week). These storms occur several times every year and some were particularly strong such as in Feb 2012. Some winters were rather mild (2008, 2014, 2016), some springs particularly stormy (2008, 2009, 2015). Over the bloom region, and from 1998 to 2017, the relative proportion of 8-day bins during which a positive variation in NHF (or a negative variation in WS) was associated with a positive variation in SChl or vice-versa was around 70% in winter and 55% in spring (Fig. 1e,f).

We examined separately three types of situations which we distinguished based on the sign of the NHF during two consecutive 8-day time steps (Fig. 3b–d): (1) the unstable winter situation, when active turbulent mixing takes place (NHF are negative during the first 8-day time step and remain negative during the following time step), (2) the transition phase, when convection shuts down (NHF are negative during the first time step and positive during the second time step) or when convection resumes (NHF are positive during the first time step and switch to negative during the second time step) and (3) the stable spring situation, when mixing is weak (NHF are initially positive and remain positive). There were several years (for instance 2008, 2010 and 2013) where the NHF oscillated between positive and negative values before transitioning to positive. During such years, in Fig. 3a, we only accounted for the last zero-crossing to test the convection shutdown hypothesis, as in Ferrari et al.^13^. In Fig. 3b**,** which shows the net growth rate against the time derivative of the NHF during the transition period, we accounted for all zero-crossing events. This enabled us to extend the initial concept of the convection shutdown hypothesis to weekly fluctuations. We recall that by definition, during the transition period, the NHF at two consecutive time steps have opposed signs. Thus by construction in Fig. 3b, positive zero-crossings are on the right quadrant (i.e. NHF switches from negative to positive indicating a positive time derivative) and negative zero-crossing on the left quadrant of the panel (i.e. NHF switches from positive to negative corresponding to a negative time derivative). An important result for the transition period is that positive zero-crossings are always associated with positive net growth rates, showing that the convection shutdown hypothesis not only applies to the main period of net positive growth (last zero-crossing from negative to positive), but also to all subseasonal events that occur before the NHF definitely turns positive. In addition, negative zero-crossings are associated with negative net growth rates and the strength of the net SChl growth is, to a large extent, proportional to the time derivative in NHF (more specifically to changes in latent and sensible heat flux, Supplementary Fig. S2). Because of the strong correlation (~ − 0.8) between weekly changes in NHF and WS (Fig. 2), a similar relationship is found when using the rate of change in wind stress (Supplementary Fig. S2). With our initial assumption that during winter and early spring, the rate of change in NHF measures the change in vertical stability, these results show that during the onset phase of the bloom, subseasonal fluctuations in SChl are driven by the intermittency in vertical stability: when NHF decrease, vertical stability decreases and SChl decreases, and vice versa. A similar relationship is found during the unstable winter situation, with net positive growth when NHF increase and net negative growth when NHF decrease (Fig. 3c and Supplementary Fig. S2). We can note that in winter the slope is flatter, illustrating that changes in vertical stability have a weaker impact on the net growth rate when the background situation is already strongly unstable. In contrast, during the mature phase of the bloom (Fig. 3d), fluctuations in net SChl growth rates are only connected to negative changes in NHF; unlike during more unstable periods, a positive change in NHF which causes even more stratification is not associated with net growth. Another interesting difference is that the NHF derivative and the net growth rates remarkably cross at the 0.0 point in winter and transition periods, but not in spring. This indicates that net growth is close to zero in winter and early spring in the absence of significant variations in atmospheric forcing, but in spring it is on average negative independently of the external atmospheric forcing. This is suggestive that mechanisms internal to the ecosystem (such as grazing) exert a significant control on SChl in spring.

## Discussion

The Northwestern Mediterranean Sea bloom shares many characteristics with the North Atlantic spring bloom^21^, with the particularity that its spatial extension is constrained to the area of winter deep convection, in the center of the cyclonic circulation of the Ligurian Sea (Fig. 1). Early modelling studies of the water column^28^ and subsequent in-situ observations^6,23^ have suggested that, as for the North Atlantic, the seasonal accumulation in SChl resulted from the alleviation of light limitation on phytoplankton growth, triggered by the reduction in vertical mixing associated with the cessation of deep convection. In light of these earlier studies, we revisited the link between vertical stability and phytoplankton accumulation using two decades of ocean color data confronted to two decades of atmospheric reanalysis. Our results support the convection shut down hypothesis^10^ which states that the seasonal surface bloom is initiated by the shut down of vertical mixing induced by the seasonal change of sign in the NHF. We should note that this hypothesis does not seem to apply universally to all bloom regions of the world's ocean. It was clearly demonstrated in the North Atlantic^13,20^ but was less convincing in the North Pacific and Southern Ocean^29^. Other studies have reported that a reduction in wind speed may also lead to a drop in turbulent mixing and cause the bloom onset, for instance around New Zealand and in the Irminger Basin^18,30^.

The seasonal paradigm explains the variability in the timing of bloom initiation, which is well constrained by the time at which the net heat flux switches from negative to positive and remains positive. But it is not sufficient to explain the strong year-to-year variability in bloom phenology. Here we argue that an overlooked complexity is that the bloom of the Northwestern Mediterranean Sea is largely chaotic in response to the chaotic nature of wind bursts (the so-called Mistral and Tramontane) occurring in winter and spring. In winter, the violent mixing episodes leading to deep convection generally last for less than a week, and can occur several times during the same winter with periods of calm weather in between^26,31,32^. During early spring, strong winds associated to large heat losses are able to destabilize the newly and weakly stratified water column. These storms lead to strong intermittency in vertical stability. We demonstrated that weekly intermittency in both NHF and WS were in phase with the chaotic fluctuations of SChl during periods of low vertical stability. When the NHF was negative or close to zero and the wind intermittently reduced, increases in NHF were associated with increases in SChl; when the wind increased, reductions in NHF were associated with reductions in SChl (Fig. 3). It is out of the scope of this study to precisely characterize the link between the intensity of vertical mixing and the changes in NHF and WS. Nevertheless our results suggest that changes in NHF and wind may be rapidly translated into changes in vertical mixing intensity, affecting light exposure of phytoplankton and their accumulation rates.

We found a large spread in the relationship between our indexes of vertical stability and phytoplankton net growth rates (Supplementary Fig. S2). This spread questions our hypothesis that phytoplankton net growth rate depends essentially on vertical stability. Other factors, such as variability in phytoplankton loss rates, come into play and may cause some deviation. Another reason is that, in addition to the atmospheric forcing, vertical stability can be affected by the (sub-)mesoscale circulation^33^. Particularly in this region of deep water formation, it has been shown that eddies, which participate in the restratification following deep convection, impact deep convection^34,35^, the spring phytoplankton bloom^28,36^ and can cause the bloom to start prior to seasonal stratification^37^. In the North Atlantic subpolar gyre, Lacour et al.^9^ observed transient winter blooms from autonomous bio-optical profiling floats that they attributed to intermittent restratification by mixed-layer eddies. The 8-day resolution of the satellite SChl hinders our ability to detect shifts of less than 8 days, nevertheless there were some years, like 2008, 2011 and 2014, where the increase in SChl was clearly ahead of time compared with the change of sign in NHF, supporting the hypothesis of intermittent eddy-driven stratification. Nevertheless, the strong connection between changes in net growth rates and changes in atmospheric conditions evidenced in this study (about 70% of the time in winter Fig. 1e) suggests that cessation of wind bursts plays a leading order role on driving winter bloom compared with purely oceanic eddy processes in this region.

During the mature phase of the bloom in April (Fig. 3d), phytoplankton net growth rates continued varying with large subseasonal variations, but with less systematic connections with atmospheric forcing. During this period of more steady physical conditions, other processes such as top down control or nutrient limitation come into play, and the phytoplankton phenology moves from a physical control to a stronger biological control. It is possible that subseasonal variations during this period ensue from intrinsic variability related to biological interactions, due for instance to predator–prey interactions^38^, or competition of different phytoplankton species for resources^21,39^.

We should note that previous studies that have explored the link between storminess and subseasonal fluctuations in phytoplankton were focussed on summer stable conditions, during which phytoplankton growth is limited by nutrient availablity rather than light^40–44^. In that case, storms tend to favor productivity by supplying nutrients to the otherwise depleted euphotic layer. The situation explored here shows an opposite relationship: storms are associated with reduced SChl. Two cases of weekly fluctuations emerge from our analysis. The first is the case of intermittent cessation of harsh atmospheric conditions, which allows the development of short blooms during periods of temporary aleviated light limitation (upper right quadrants in Fig. 3b,c, positive net growth and positive NHF derivative). The second is the case of storms that temporarily interrupts phytoplankton accumulation, with net growth at the surface decreasing in response to the dilution of phytoplankton by vertical mixing (lower left quadrants in Fig. 3b–d, negative net growth and negative NHF derivative).

It is important to highlight that our analysis is based on surface chlorophyll data, and that a distinction is to be made between the surface chlorophyll signal and the vertically integrated phytoplankton carbon biomass. The former is easy to routinely monitor through remote sensing and is characterized by a sharp increase in early spring when conditions become favorable. The latter is only accessible through costly and disparate in-situ observations collected from various observing means^7,45,46^ and can show an increase that starts before the surface bloom and at a slower rate when the integrated phytoplankton population growth rate and loss rate are decoupled^7,46,47^. In the case of subseasonal SChl fluctuations, one may wonder whether they reflect variations in total biomass, variations in carbon to chlorophyll ratios or essentially mirror dilution when the vertical extent of mixing varies. The field experiment carried out from July 2012 to July 2013 in the Gulf of Lion (GOL box in Fig. 1) provides some insight to these questions^6^. During the 2013 winter-spring transition, variations in particulate organic matter in the mixed-layer were monitored, and mirrored those of SChl. This observation, even if limited in time, suggests that subseasonal SChl variations can be interpreted as variations in surface carbon biomass in winter and spring. Also, interrestingly, the 2013 bloom was interrupted by a strong wind event in mid-March (Fig. 2). Temporal vertical profiles of chlorophyll observed during that event^6^ unambiguously showed that the drop in SChl was solely due to dilution of chlorophyll well below the euphotic layer. Nevertheless a lagged effect (by approximately one week) with increased integrated chlorophyll was also observed, suggesting the possibility of more complex net growth dynamics for total biomass, which may result from reduced grazing due to dilution^47^ following the wind event.

An open question is the overall importance of subseasonal events on the functioning of the system. Our analysis suggests that the brief and episodic variations in phytoplankton observed during the transition from winter to spring ensue from variations in vertical stability that modulate phytoplankton growth rates. These variations are likely to shape the composition of the entire plankton community. For instance, Lacour et al.^9^ reported a community shift from pico and nanophytoplankton to diatoms during transient winter blooms, that likely trigger a similar shift in zooplankton species. A recent analysis based on profiling float measurements in the North Atlantic also revealed short-term changes in grazing during the spring bloom transition^7^. These different manners by which subseasonal variations in vertical stability affect herbivores predation and therefore growth, are likely to influence the transfer of energy to the higher trophic levels^48^.

Finally, our results suggest that subseasonal events potentially make a significant contribution to the annual export of carbon to the ocean’s interior. First, because the community shift to larger phytoplankton species should be associated with large export through the gravitional pump^9^. Second, because intermittent storms during the bloom trigger export through dilution^6^, through the so-called mixed-layer pump^49^. Our results show that these strong events of mixed-layer pump export can be identified with adequate time-series of SChl and NHF, but vertical data would be needed to quantify the quantity of organic material being exported. Given the prevalence of SChl subseasonal events during the bloom and the important role they might have on export production and food web dynamics, more dedicated studies are needed to improve our understanding of these events and their consequences.

## Data and methods

We analysed concomitant time series of SChl and atmospheric reanalysis (NHF and WS) over the period 1998–2017 in the bloom region of the Northwestern Mediterranean sea (delimited by the black contour in Fig. 1). For SChl, we used the 8-day, 4 km × 4 km resolution, level 3 mapped ocean color product (release 3.1) distributed by the European Space Agency Ocean Color Climate Change Initiative (ESA OC-CCI) available at http://www.oceancolour.org/. Each time step represents the averaged SChl value over a period of 8 days, estimated from all available daily ocean color observations during the time period. This 8-day product has excellent data coverage in the Northwestern Mediterranean Sea^8^. We used WS and air-sea NHF from the ECMWF ERA Interim reanalysis^50^ available at daily and 0.125° × 0.125° spatial resolution from https://apps.ecmwf.int/datasets/data/interim-full-daily/. NHF were computed as the sum of the shortwave radiation, long wave radiation, latent heat flux and sensible heat flux. WS was computed from zonal and meridional surface wind components. In order to facilitate comparison between data sets, atmospheric data were averaged over the 8-day temporal grid of SChl and SChl data were interpolated over the spatial atmospheric grid. Our statistics are thus based on the comparison of SChl, NHF and WS time-series at each pixel in the bloom region, with a 8-day time resolution and 0.125° spatial resolution (a total of 394 pixels and 305 time steps for the bloom region over 1998–2017).

Vertical stability is decreased during storms in response to the mechanical action of the wind and to the loss in buoyancy that goes with it. We used time derivatives of WS and NHF as a proxy for intermittent changes in vertical stability. Strong positive changes in WS (d(WS)/dt > 0, corresponding to d(NHF)/dt < 0) indicated the passage of storms, while negative changes in WS (d(WS)/dt < 0, corresponding to d(NHF)/dt > 0) marked the return to low wind conditions. In this study, results are shown using d(NHF)/dt; the corresponding analysis using d(WS)/dt is provided in thesupplementary material.

Phytoplankton net growth rates (in units of day^−1^) are computed as the time derivative of SChl in log scale, d(ln(SChl)/dt). All time derivatives (Net growth, WS, NHF) are computed between two consecutive 8-day time steps.

All the figures in this manuscript are generated using SAXO (http://forge.ipsl.jussieu.fr/saxo/download/xmldoc/whatissaxo.html) based on IDL-6.4 (Interactive Data Language).

## Supplementary Information

Supplementary Information.
