# Supplementary material for: Intermittency in phytoplankton bloom triggered by modulations in vertical stability
Source: Sci Rep. 2021 Jan 14;11:1285. doi: 10.1038/s41598-020-80331-z (PMC7809256; doi:10.1038/s41598-020-80331-z)
Supplement: Supplementary file 1 — Supplementary Information. [file 41598_2020_80331_MOESM1_ESM.docx]

**Intermittency in phytoplankton bloom triggered by modulations in vertical stability**

M. G. Keerthi^1*^, M. Levy^1^, and O. Aumont^1^

^1^ Sorbonne Université (CNRS/IRD/MNHN), LOCEAN-IPSL, Paris, France

***Corresponding author: Keerthi M G (keerthi.madhavan-girijakumari@locean.ipsl.fr)**

**Contents of this file**

Figure S1, S2


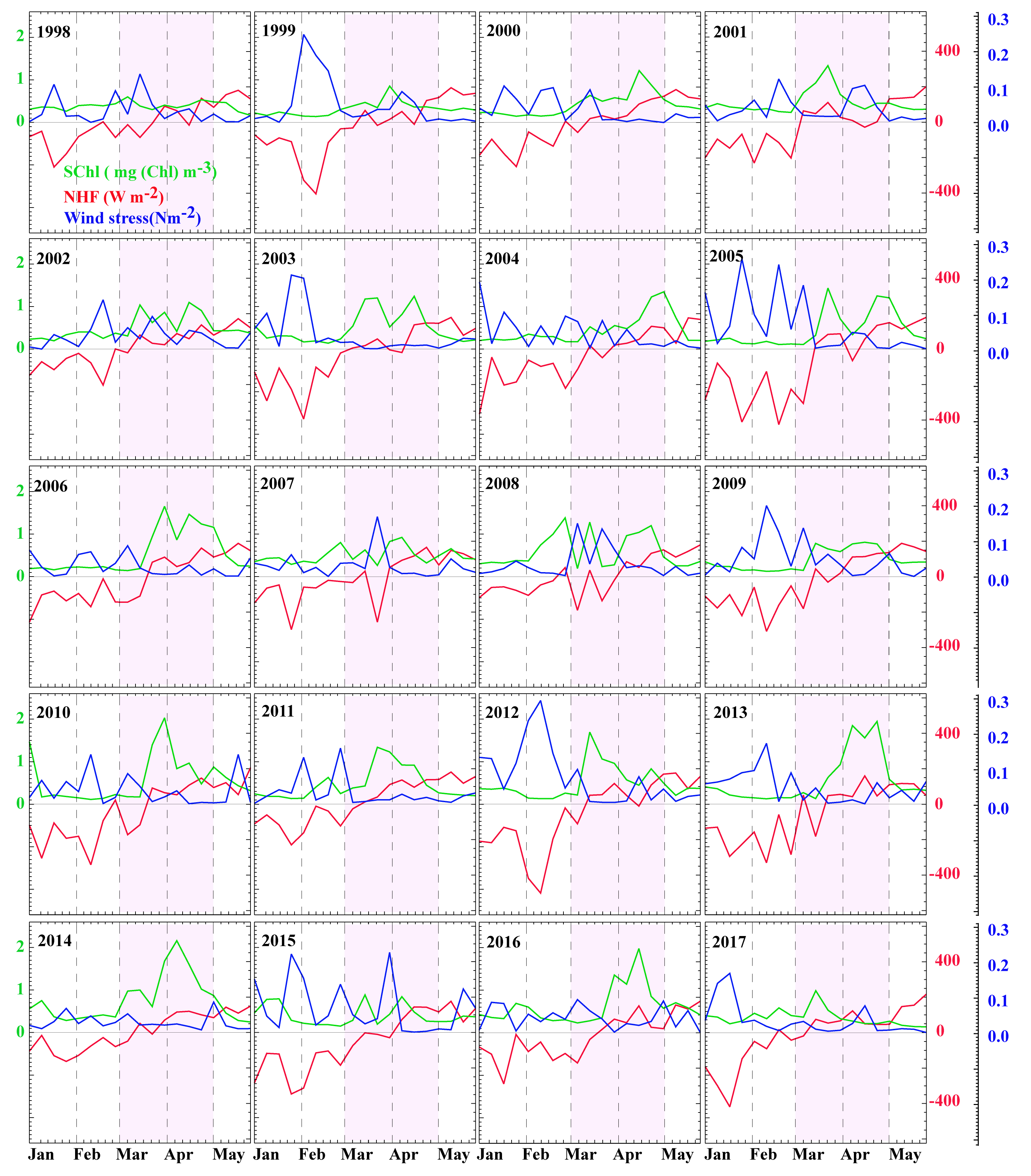


**Supplementary Figure S1:** Timeseries of Surface Chlorophyll (SChl, green curves), Net Heat Flux (NHF, red curves) and wind stress (blue curves) averaged over the GOL box between January and May, from 1998 to 2017. The pink shading highlights the spring period (March-April).


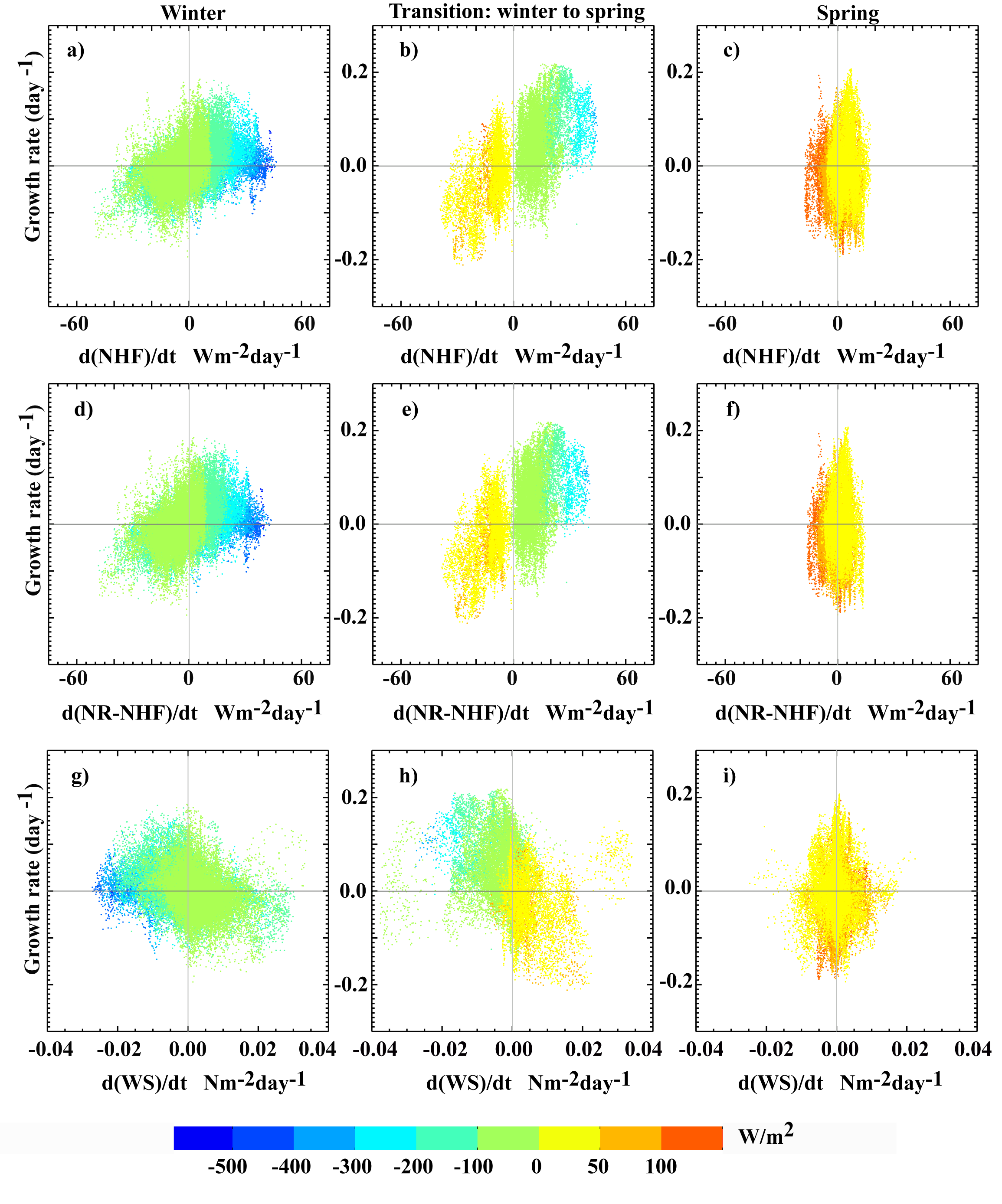


**Supplementary Figure S2:** Scatter plot of SChl net growth rate versus temporal changes in Net Heat Flux - d(NHF)/dt (first row), Non radiative Heat flux - d(NR-NHF)/dt (second row) and wind stress - d(WS)/dt (third row) during three phases of surface stability, **(a, d, g) the unstable winter phase:** when NHF are initially negative and remain negative **(b, e, h) the transition phase:** when NHF is initially negative and switches to positive; or when NHF is initially positive and switches to negative **(c, f, i) the stable spring phase:** when NHF is initially positive and remains positive**.** Each dot corresponds to individual events, i.e. at each 8-day time step and at each 0.125° x 0.125° pixel in the bloom region within the time period January-April, over years 1998 to 2017. The color of the dots shows the value of the NHF at the beginning of the 8-day time period. In b), the net growth rates are negative when the NHF are initially positive (warm colors in the lower left quadrant) and switch to negative. They are positive when the NHF are initially negative (cold colors in the upper right quadrant) and switch to positive; we can note that the positive net growth rates tend to be relatively weaker for initially strongly negative values of the NHF.
